# Crohn’s Strictures—Moving Away from the Knife

**DOI:** 10.3389/fped.2017.00141

**Published:** 2017-06-16

**Authors:** Emily Stenke, Billy Bourke, Ulla Knaus

**Affiliations:** ^1^School of Medicine, Conway Institute, University College Dublin, Dublin, Ireland; ^2^Department of Pediatric Gastroenterology, Our Lady’s Children’s Hospital, Dublin, Ireland

**Keywords:** Crohn’s disease, inflammatory bowel disease, fibrosis, intestine, reactive oxygen species, NADPH oxidase

## Abstract

Crohn’s disease (CD) is a lifelong inflammatory bowel disease with a rapidly rising incidence in the pediatric population. A common complication of CD is the development of fibrotic strictures, which may be present at initial diagnosis or develop many years later. Clinical presentation depends on stricture location and degree of obstruction, and strictures frequently contain a mixture of inflammatory and fibrotic tissue. Histological examination of Crohn’s strictures shows thickening of the muscular layers and the submucosa, where increased collagen deposition by activated myofibroblasts is concentrated around islands of smooth muscle cells and at the superficial margin of the muscularis propria. No antifibrotic therapies for Crohn’s strictures exist. Profibrotic transforming growth factor-β (TGFβ)/bone morphogenetic protein signaling stimulates myofibroblast differentiation and extracellular matrix deposition. Understanding and targeting TGFβ1 downstream signaling is the main focus of current research, raising the possibility of specific antifibrotic therapy in CD becoming available in the future.

## Introduction

The incidence of Crohn’s disease (CD) is rapidly rising in the pediatric population (1). Crohn’s inflammation is transmural and can occur throughout the gastrointestinal tract, unlike ulcerative colitis where mucosal inflammation is confined to the colon. CD is phenotyped according to disease behavior (inflammatory/penetrating/stricturing) and location using the Paris classification for pediatric patients (2), a modification of the Montreal classification (3). Fibrotic narrowing of the intestinal tract (stricturing disease) is present in approximately 10–17% of children at diagnosis (4), affecting up to 40% by 10 years after diagnosis (5–7). Strictures occur predominantly in the ileum, reflecting the common distribution of inflammation, but can arise throughout the digestive tract (8). Presenting features vary from overt intestinal obstruction to subacute and non-specific symptoms. The conventional conceptual framework of the pathobiology of fibrosis in CD is one of chronic inflammation leading to ongoing frustrated attempts at healing with formation of disorganized tissue. If this occurs circumferentially in a relatively narrow diameter organ such as the small intestine, the result is a fibrotic stricture. Here, we will describe current knowledge and recent advances regarding the diagnosis and pathogenesis of intestinal fibrosis and will review potential future therapies.

## Clinical and Pathological Diagnosis

The clinical presentation of stricturing disease is highly variable, depending on the location and degree of obstruction. Presentations range from non-specific symptoms including abdominal discomfort, poor appetite, energy and/or growth, to overt obstruction with abdominal pain, vomiting, and reduced bowel movements. Strictures may be inflammatory, fibrotic, or more commonly a combination of both (8). Most strictures are in the small bowel, inaccessible to standard endoscopy, and even when accessible, endoscopy can only provide information (visual, histological) on the mucosa, while the fibrotic collagen deposition occurs submucosally. Capsule endoscopy can provide visual information but no tissue samples and is contraindicated in the presence of suspected strictures due to the risk of retention. Therefore, clinicians usually rely on radiological tools for the diagnosis of stenotic disease.

A fibrotic stricture is inferred by the presence of a narrowed lumen with proximal dilation. Computed tomography (CT) and magnetic resonance enterogram (MRE) are preferred to X-rays with enteral contrast [small-bowel follow through (SBFT)] as they allow assessment of extraintestinal as well as intestinal complications of CD and have similar sensitivity and accuracy. CT and MRE permit a transmural assessment of the bowel wall, enabling the classification of strictures as inflammatory, fibrotic, or mixed. Inflammation is suggested by avid enhancement and mesenteric inflammation (hypervascularity, fat stranding), whereas fibrosis is characterized by a thickened bowel wall with a featureless appearance, minimal or no enhancement and the absence of mesenteric inflammation. MRE has comparable (9–12) or superior (13) sensitivity and specificity to CT for the detection of fibrosis in the presence of inflammation in adult and pediatric patients, while MRE has greater sensitivity for the detection of fibrosis alone (10). A recent prospective pediatric study demonstrated 73% sensitivity and 81% specificity for the detection of strictures using MRE compared to 42% sensitivity and 68% specificity for CT enterograms (12). Due to its lack of ionizing radiation and its capacity to provide accurate diagnosis of both intra- and extraintestinal complications, MRE is the preferred modality in the pediatric population, although technical limitations (institutional access, need for general anesthesia) ensure a retained role for CT and SBFT. Recent advances in ultrasound elastography as a measurement of tissue stiffness have shown promising results in the differentiation between inflammatory and fibrotic strictures (14, 15), but have not yet reached routine clinical practice. As sensitivity does not reach 100% with any modality, clinical judgment is required in conjunction with imaging findings to decide about surgical exploration/intervention.

## Histopathology

Fibrosis is defined as the permanent and abnormal deposition of extracellular matrix (ECM), primarily collagen, within tissues, resulting in a distortion of structure and impeding normal tissue and organ function. It is understood to be an aberrant response to ongoing inflammation, where tissue remodeling in response to injury, through ECM deposition and subsequent breakdown, becomes self-perpetuating. In the normal small bowel, the mucosa consists of a single layer of epithelium, lamina propria, and basement membrane. Deep to the intact mucosa is the muscularis mucosa, a thin layer of smooth muscle cells, and then the submucosa. The submucosa is a loose connective tissue layer with fibroblasts as main cell type within an ECM traversed by blood vessels and nerves (16). The ECM is a complex biochemical structure where collagen types I and III predominate (17). Deep to the submucosa is the muscularis propria with its inner circular and outer longitudinal layers of smooth muscle cells. The collagen strands of the submucosa tend to be concentrated at its border with the muscularis propria.

**Figure 1:**
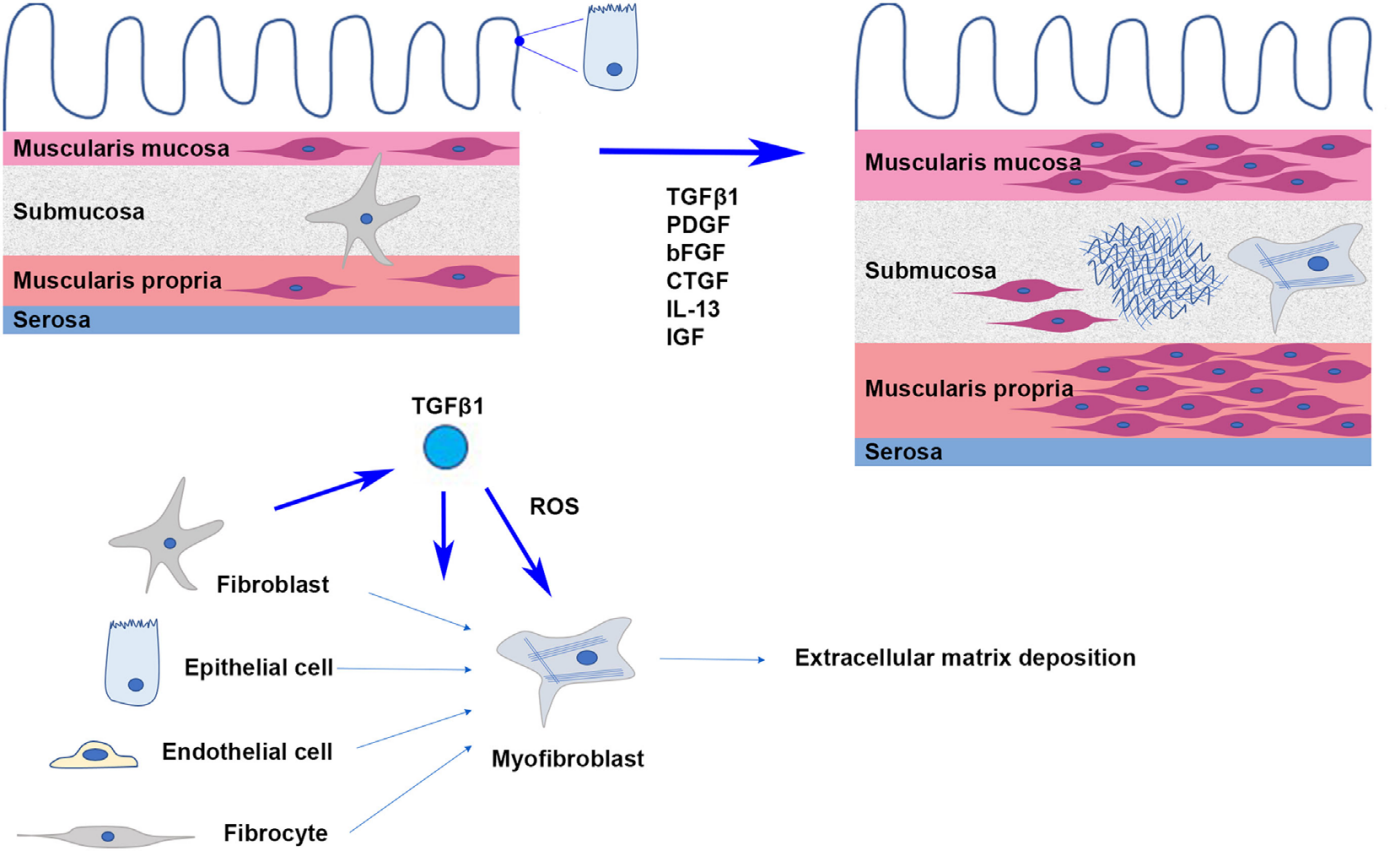
In intestinal fibrosis, hypertrophy and hyperplasia of smooth muscle cells cause thickening of the muscularis mucosa and muscularis propria; smooth muscle cells proliferate in the thickened submucosa, where activated myofibroblasts secrete extracellular matrix proteins.

Histological examination of CD strictures reveals abnormalities in both the submucosal space and muscular layers (Figure 1). The submucosa is increased in volume and density, with increased collagen deposition being concentrated around islands of smooth muscle cells and at the superficial margin of the muscularis propria. Studies have shown an increase in total protein and collagen content, especially collagen subtypes I, III, and V in these strictures. Although increased in absolute terms, the relative proportions of types I and III collagen appear to be comparable between strictures and healthy intestine, and although the proportion of type V collagen is relatively amplified, type I collagen remains dominant (16, 17). Myofibroblasts, differentiated from fibroblasts, are the primary source of ECM production and secretion. Increased numbers of local fibroblasts and myofibroblasts in fibrosis have been attributed to a variety of mechanisms including the proliferation of existing fibroblasts in the local area (18), the induction of epithelial-to-mesenchymal transition (19), the recruitment and differentiation of bone marrow-derived fibrocytes (20), as well as endothelial-to-mesenchymal transition (21), but the relative contribution of each process is unknown. Thickening of the muscularis mucosa and muscularis propria as well as smooth muscle cell proliferation within the submucosa itself have been observed. In some instances, the proliferation of smooth muscle cells within the submucosa can be so pronounced that it results in the obliteration of the submucosa (22). The combined overall effect of increased ECM deposition and muscular hypertrophy is transmural thickening and stiffening, which when occurring circumferentially causes narrowing and obstruction of the intestinal lumen.

## Signaling and the Fibrotic Response

Various growth factors and cytokines have been implicated in the development of fibrosis including IL-13, platelet-derived growth factor (PDGF), connective tissue growth factor, basic fibroblast growth factor, insulin-like growth factor, bone morphogenetic proteins (BMPs), and transforming growth factor-β (TGFβ) (23). The TGFβ family is secreted by a wide variety of cells throughout the body and its effects are highly varied and complex. As well as being responsible for cell proliferation and differentiation, anti-inflammatory control, wound healing, tumor suppression in healthy tissues and cancer progression in neoplastic tissues, TGFβ is the primary cytokine driving the development of fibrosis in tissues throughout the body. Differences in TGFβ subtype, receptors, cofactors, and signaling pathways allow this group of cytokines to display such versatility (24). TGFβ1 is the most common subtype and its role in the development of tissue fibrosis through the recruitment of fibroblasts, transdifferentiation to myofibroblasts, and stimulation of ECM secretion has been convincingly demonstrated in many organs including in intestinal fibrosis (25–28).

**Figure 2:**
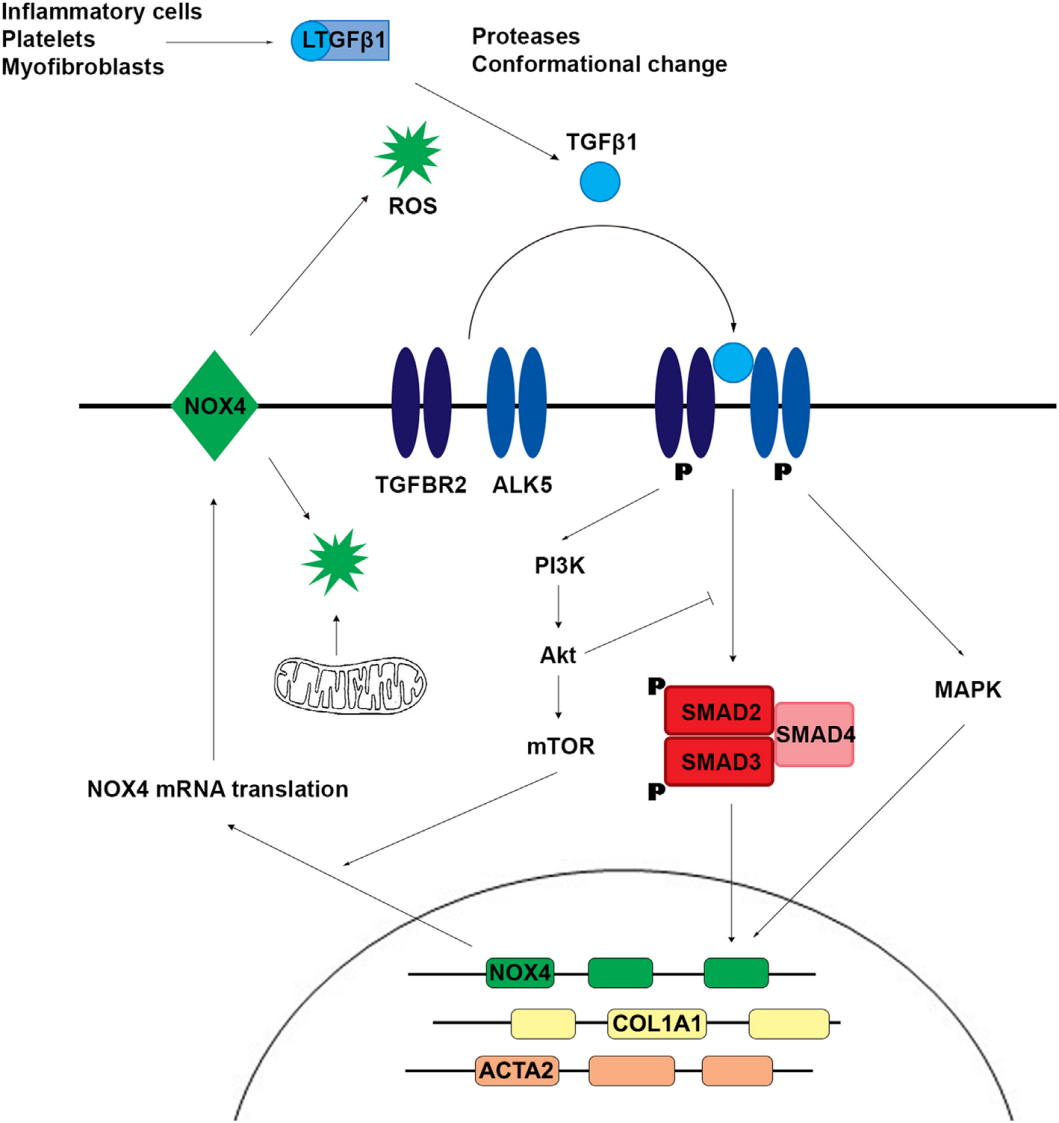
TGFβ1 signaling induces SMAD2 and SMAD3 phosphorylation and activation of non-canonical mitogen-activated protein kinase (MAPK) and phosphoinositide 3-kinase (PI3K) pathways, which upregulate the expression of profibrotic genes including NADPH oxidase 4 (NOX4). Reactive oxygen species (ROS) generated by NOX4 and/or mitochondria promote further release of active TGFβ1.

TGFβ1 is a homodimeric signaling molecule produced by myofibroblasts and inflammatory cells (e.g., macrophages), platelets, and parenchymal cells during the hemostasis and inflammation phases of tissue injury and healing. This para- and autocrine signaling is anti-inflammatory and supports wound healing but drives fibrosis. The TGFβ receptor is a transmembrane complex of two dimers: two type I receptors (ALK5, also called TGFBR1 or TβTI) and two type II receptors (TGFBR2); both are serine/threonine kinases and binding of TGFβ results in phosphorylation of ALK5 by TGFBR2 with subsequent binding and phosphorylation of downstream signaling proteins SMAD2 and SMAD3 by ALK5 (Figure 2). Once phosphorylated, SMAD2 and SMAD3 form a trimer with SMAD4, which translocates to the nucleus and binds to target DNA. The transcriptional targeting, nuclear translocation, and longevity of these SMAD transcription factors is modulated by the binding of a wide variety of effector molecules. SMAD2/3 binding to ALK5 is antagonized by SMAD7. SMAD7 antisense oligonucleotides (mongersen) showed promise in the treatment of inflammatory CD by restoring the TGFβ1–SMAD2/3 pathway (29); no associated increase in fibrosis has been observed but longer term follow-up is required. Several non-canonical TGFβ signaling pathways have been described (30). Phosphorylated TGFBR1 and TGFBR2 lead to activation of mitogen-activated protein kinases (MAPKs), which regulate multiple pathways including those leading to transcription. MAPKs also regulate the canonical TGFβ pathway by phosphorylating active SMAD to promote its proteasomal degradation. TGFβ activates phosphoinositide 3-kinase, leading to downstream activation of Akt, mTOR, and upregulation of protein translation. Akt interacts directly with SMAD3 to prevent its activation and indirectly inhibits SMAD-mediated transcription through phosphorylation of FoxO transcription factors, thus blocking their translocation to the nucleus. A more detailed review of the complexity of TGFβ signaling can be found in the study by Massague (24).

The pleiotropic and multifunctional effects of TGFβ1 signaling make it an unattractive therapeutic target in fibrosis. Substantial effort has been directed at characterizing downstream signaling pathways of TGFβ where fibrosis-specific pharmacologic intervention might be achievable. Reactive oxygen species (ROS) have been implicated as mediators of the profibrotic effect of TGFβ1. ROS can be produced non-enzymatically (e.g., by radiation, toxic chemicals) or enzymatically. Most cellular ROS is produced in the mitochondria during cellular respiration. Other ROS sources include the cytochrome P450 family, cyclooxygenases, peroxisomal oxidases, xanthine oxidoreductase, and the family of NADPH oxidases (NOX). The NOX family is unique as its only known biological role is ROS production, and NOX enzymes appear to be intimately involved in fibrogenesis. NADPH oxidase 4 (NOX4) is a constitutively active, transcriptionally regulated producer of H_2_O_2_. An accumulating body of evidence suggests that TGFβ1 upregulates NOX4 expression and that NOX4, through the production of H_2_O_2_, promotes myofibroblast differentiation and secretion of ECM proteins (31–33). In a rat model of renal fibrosis and in cell-based assay systems, TGFβ1 stimulation was associated with increased NOX4 expression and H_2_O_2_ production, while reduced NOX4 expression by siRNA-mediated knockdown decreased ROS production and expression of profibrotic proteins including collagen, α-SMA, and fibronectin (34). These findings have been replicated using cell-based assays, mouse models, and patient samples in the lung (35–38), skin (39), and liver (40–42). A study by Jung et al. using fibroblasts and a mouse model of renal fibrosis suggested that NOX4 upregulation by TGFβ1 depends on activation of SMAD2 and Akt (43). Latella and coworkers reported that SMAD3 was necessary for the development of intestinal fibrosis in a chronic TNBS model (44), suggesting that NOX4 mediates the effect of TGFβ1 through canonical and non-canonical pathways. Somewhat contradictorily, NOX4 was protective in a renal fibrosis model (45), and there is evidence from mice and patient data that NOX4 may be protective against atherosclerosis (46–49). Another mouse model showed preserved myofibroblast differentiation but impaired wound healing in NOX4−/− mice (50), suggesting that the requirement of NOX4 for myofibroblast differentiation and collagen secretion may be tissue and/or context dependent (acute versus chronic insult). Despite the growing evidence for a fundamental role of NOX4 in fibrosis, only limited studies exist for the intestine. Hotta and coworkers demonstrated a reduction in TGFβ1-dependent collagen I production by murine intestinal myofibroblasts treated with a pan-NOX inhibitor or NOX4 siRNA (51). Data from RNA-Seq analysis of intestinal fibroblasts showed variable upregulation of NOX4 transcription in three patients with CD compared to three healthy controls (52). BMPs belong to the TGFβ superfamily and signal *via* phosphorylation and complex formation of SMAD 1, 5, and 8 (53). BMP7 may protect against colitis and prevent fibrosis by antagonizing TGFβ signaling (54–56). Angiopoietin-like protein 2 (ANGPTL2) modulates BMP signaling and initial studies suggest that organ damage in ANGPTL2 knockdown mice is linked to NOX4 (57, 58).

TGFβ1 signaling stimulated the production of mitochondrial ROS (mtROS), possibly by inhibition of complex III and IV and *via* the mTOR signaling pathway, with subsequent increase in profibrotic gene expression. Reduction of mtROS by the antioxidant MitoQ reduced TGFβ1 expression, SMAD2 and SMAD3 activation, and collagen deposition in a liver fibrosis model (59). mtROS and NOX4 may interact through a positive feedback loop to promote TGFβ1-driven fibrosis. NOX4-derived ROS caused mitochondrial dysfunction and increased mtROS, while mtROS amplified the TGFβ1-mediated increase in NOX4 expression (60). Additionally, increased TGFβ1 inhibited the antioxidant response, thereby exacerbating the prooxidant shift and further driving fibrosis (61).

Key enzymes in cross-linking and stabilizing the network of collagen fibrils are H_2_O_2_-generating lysyl oxidase (LOX) and the lysyl oxidase-like proteins (LOXL1–4), which are copper-dependent amine oxidases that oxidatively modify the ε-amino group of lysine side chains in collagen and elastin for formation of inter- and intrachain cross-links. Clinical and animal-based studies in the liver and myocardium demonstrate that LOXs promote tissue stiffening through cross-linking of existing collagen and elastin fibrils and that inhibition of LOXL2 may inhibit and even reverse fibrosis (62–65). Studies in rat and human lung fibroblasts, human trabecular cells, and human osteoblasts suggest that TGFβ1 upregulates LOXs, which in turn modify the actions of TGFβ1 (66–69). It is important to note that TGFβ1 is secreted and stored extracellularly bound to a latent TGF-β-binding protein and a latency-associated peptide. This inactive complex is bound to the ECM *via* integrins, and active TGFβ is released by protease cleavage or conformational changes caused by increased stiffness of the ECM (61, 70). This provides a possible explanation for TGFβ1’s effects in the absence of active inflammation (71, 72) as well as the finding that a stiff matrix is required for myofibroblast differentiation (62, 73).

## Current Treatments and Future Possibilities

Although improved management of CD inflammation by anti-TNFα therapy (infliximab, adalimumab) appears to reduce the rate of stricture development (74), there is currently no medical therapy directly targeting fibrosis in CD. Patients whose strictures fail to respond to anti-inflammatory therapies (aimed at any inflammation and edema coexisting with fibrosis) require surgical intervention. Endoscopic balloon dilatation (EBD) is an option for single, short, and uncomplicated strictures accessible by endoscopy, for instance, stricture recurrence at ileocecal anastomoses. Although technical success rates are high, with a low rate of complications, retrospective data from adult patients demonstrate that 42–70% of patients will require repeat EBD or surgical intervention by 5 years (75–77). Pediatric data are limited, but support the feasibility and safety of EBD (78, 79). Given the predominance of mixed fibrotic/inflammatory strictures over purely fibrotic strictures, intrastricture injection of corticosteroids has been proposed as an adjunct to balloon dilation. A prospective randomized control trial including nine adult patients showed no difference in stricture recurrence rates at 1 year (80), while a prospective RCT including 29 pediatric patients showed earlier stricture recurrence in patients treated with placebo (79). Due to the small trials and number of patients studied, the benefit of intralesional steroid injection has not been confirmed (81). Surgery is the mainstay of treatment for fixed Crohn’s strictures. Simple, short strictures can be treated with bowel preserving strictureplasty; longer, multiple, or complicated strictures (e.g., significant inflammation, penetrating disease, and suspected cancer) are treated with resection and primary anastomosis. Resection carries its own risks, including anastomotic dehiscence and disease recurrence at the site of the anastomosis, malabsorptive issues following terminal ileal resection including vitamin B12 deficiency and bile salt malabsorption, and, in cases requiring repeated resections for recurrent strictures, short bowel syndrome leading to a dependence on parenteral nutrition.

Medical therapies to prevent and reverse fibrosis are eagerly sought and much of the focus for new therapies has been for pulmonary and hepatic disease. As our understanding of the pathophysiology of fibrosis improves, we are discovering more potential drug targets. Pirfenidone, a growth factor inhibitor, has been licensed for use in idiopathic pulmonary fibrosis (IPF), based on positive results from Phase 2 and 3 trials (82, 83). Nintedanib, a kinase inhibitor that acts on vascular endothelial growth factor receptors, PDGF receptors, and fibroblast growth factor receptors, showed efficacy and safety in Phase 2 and 3 trials and is licensed for use in IPF (83). Specific NOX4 inhibitors are still not available, albeit a NOX inhibitor (GKT137831) performed well in preclinical models (35, 42) and a Phase 1 clinical trial (84). A Phase 2 trial confirmed safety but not efficacy in the treatment of diabetic kidney disease; recruitment for a Phase 2 trial in patients with primary biliary cirrhosis is ongoing. Integrin αvβ6 is another target of interest; it mediates the conformational release of active TGFβ from its latent complex (72) and Phase 2 trials of a monoclonal antibody in the treatment of IPF are ongoing (85). Given the complex interaction between TGFβ secretion and tissue stiffness mediated by myofibroblast contraction, ECM deposition, and cross-linking with further TGFβ release, it is possible that the effective treatment of fibrosis will require combination therapy. For example, both Simtuzumab, a monoclonal antibody to LOXL2, and Relaxin (an inhibitor of myofibroblast contraction) have individually lacked efficacy for fibrotic disease in Phase 2/3 trials (86). However, a recent preclinical trial demonstrated a reduction in airway fibrosis using Relaxin and anti-LOXL2 antibody together (87).

Clinical trials assessing antifibrotic efficacy of currently available drugs in CD have not yet been initiated. Three times daily oral doses of pirfenidone commenced at time of transplantation and continued for 7 days reduced TGFβ expression and intestinal fibrosis in an intestinal transplant mouse model (88), suggesting that it may prevent fibrosis in CD. Animal models of intestinal fibrosis will provide an opportunity for preclinical testing of future drugs that target signaling pathways (23, 89), and therapies to reverse as well as prevent fibrosis will be required (35). A separate but related challenge still to be addressed will be the development of biomarkers for the accurate categorization of patients at risk of, or in the early stages of fibrosis, in order to intervene in CD at an early stage with existing or future antifibrotic drugs.

## Author Contributions

ES: conception and drafting of article. ES, BB, and UK: revision and final approval of article.

## Conflict of Interest Statement

The authors declare that the research was conducted in the absence of any commercial or financial relationships that could be construed as a potential conflict of interest.
